# Beyond retention: an ecological analysis of teacher commitment and agency in rural China

**DOI:** 10.3389/fpsyg.2026.1790474

**Published:** 2026-06-04

**Authors:** Jianwei Wei, Sheng Wang, Sijie Fan

**Affiliations:** 1 College English Teaching Department, Guangdong University of Foreign Studies South China Business College, Guangzhou, China; 2 School of Education, Guangdong University of Foreign Studies South China Business College, Guangzhou, China; 3 Guangdong-Hong Kong-Macau Great Bay Area Shipping Research Institute, Guangzhou Maritime University, Guangzhou, China

**Keywords:** ecological systems theory, professional resilience, rural education, teacher agency, teacher commitment

## Abstract

**Introduction:**

Retaining teachers in under-resourced rural contexts is a persistent global challenge that hinges on complex psychological and ecological processes. While macro-level policies emphasize rural revitalization and social sustainability, the micro-level psychological mechanisms that sustain teacher commitment remain underexplored.

**Methods:**

Integrating Ecological Systems Theory and the Three-Component Commitment Model, this study utilizes Interpretative Phenomenological Analysis to compare the professional trajectories of “stayers” and “leavers” in rural China.

**Results:**

The findings reveal that despite sharing structural constraints such as low salaries, the two groups navigate distinctly different ecological pathways. Stayers develop a psychological “buffering effect” against environmental stressors through meso-level social inclusion and micro-level professional agency enacted via place-based education. These interactions foster deep normative and affective commitment, respectively. In contrast, leavers experience “ecological stagnation”, characterized by social exclusion and suppressed agency, leading to professional burnout and commitment breakdown.

**Discussion:**

This study suggests that enhancing the professional resilience of rural teachers requires an ecosystem that satisfies their psychological needs for relatedness and autonomy, rather than merely providing material incentives. These findings offer a novel theoretical framework for understanding the dynamic interaction between teacher psychology and the rural social environment.

## 1 Introduction

Rural areas globally are undergoing profound socioeconomic transformation. During this process, achieving social sustainability has become a highly challenging core objective, which involves maintaining the sustained welfare of rural communities through social inclusion, cohesion, and cultural transmission (Sarfo et al., 2025). The achievement of this broader goal depends heavily on a stable and psychologically resilient teaching force, considering that education is essential to maintaining community vitality (Themane and Thobejane, 2019). China offers a particularly illustrative case of this dynamic. Following the elimination of absolute poverty, the national strategic focus shifted in 2021 to rural revitalization (Li and Xue, 2021). However, amidst rapid urbanization, rural areas face profound social transformations, including population outflow and hollowing out, which create significant stressors for local educators (Wen et al., 2023). In this context, teachers function not merely as instructional staff but as essential agents bridging schools and communities (Reid et al., 2010; Barnes et al., 2024). Therefore, retaining rural teachers is no longer just an educational human resource issue. Instead, it constitutes a crucial link in achieving broader social sustainability. Understanding the psychological mechanisms that enable teachers to maintain commitment amidst these challenges is crucial for educational equity and quality.

Although the pursuit of social sustainability in rural regions requires a stable teaching force, rural teacher retention remains a complex psychological and ecological dilemma. Many policy interventions risk failure because they overlook the inherent logic of the rural social ecosystem and the specific psychological needs of teachers within it (Rongna and Sun, 2022). While existing research has extensively explored the external challenges facing rural teachers, significant gaps remain in explaining the dynamic formation of teacher commitment. Predominant research employs quantitative paradigms focused on static variables, such as well-being or self-efficacy, to fully capture the evolving psychological processes teachers experience as they interact with complex ecosystems, especially those involving local communities and cultural traditions at micro and meso levels (Zhi et al., 2025). Furthermore, current literature often focuses on single groups, highlighting a need for comparative research between “stayers” and “leavers” to understand the divergent cognitive and emotional paths of professional retention (Gao et al., 2025).

To address these gaps, this study uses social sustainability as an overarching context to construct an integrated framework that combines Ecological Systems Theory (EST) with the Three-Component Commitment Model (TCM). By comparing the lived narratives of teachers who stay and those who leave, it aims to reveal how the ecosystem shapes various dimensions of psychological commitment, and through what proactive practices teachers reshape their professional identity in ecological interactions. Specifically, this study addresses two core questions:

1. How do the lived experiences of ecological constraints and supports differ between “stayers” and “leavers,” and how do these differences account for their divergent professional trajectories?
2. How do rural teachers perceive and negotiate the interactions between different ecological systems, and through what mechanisms do these interactions shape their multidimensional commitment?

## 2 Literature review

### 2.1 Social sustainability and the role of rural teachers

Social sustainability provides the broader context for understanding contemporary changes in rural education. In the background of educational development, social sustainability is defined as the capacity to maintain the long-term well-being of a community through social inclusion, cohesion, and educational interventions (Sarfo et al., 2025). Within this framework, rural teachers serve not only as transmitters of knowledge, but also as key drivers who resist rural social decline, promote community revitalization, and advance social justice (Themane and Thobejane, 2019). However, rural education often faces inherent contradictions in achieving social sustainability. Historically, rural schools have been viewed as ladders for social mobility, acting as a sorting mechanism that often leads academically successful students to leave their hometowns, which exacerbates the brain drain in rural areas (Bæck, 2016). This suggests that if teachers merely play the role of traditional disseminators of knowledge focused on examinations, they may inadvertently weaken local social vitality. Therefore, in the contemporary rural context, the role of teachers within rural social spaces is particularly crucial and nuanced. They are expected to improve the academic performance of students while also acting as bridges that connect schools and communities to promote local sustainable development (Leach and Bradbury, 2024).

Nevertheless, the ability of teachers to effectively fulfill this function largely depends on the provision of sufficient support and recognition within their social ecosystem. Drawing on Honneth’s theory of recognition, analysis reveals that although small rural schools often face material resource limitations, the close interpersonal networks characteristic of this environment can foster solidarity relationships based on care and respect (Cuervo, 2025). This social esteem from community members and colleagues forms the psychological foundation of teacher job satisfaction. In contrast, the absence of such a supportive environment significantly weakens teachers’ sense of professional efficacy. For instance, empirical research on rural schools in Romania confirms that authentic leadership by principals positively predicts teacher self-efficacy (Baștea et al., 2024), and administrative support can effectively alleviate feelings of isolation (Toman and Maag, 2024). Therefore, social inclusion and support networks serve as a prerequisite for teachers to fulfill their role in social sustainability, and this socio-ecological attribute directly points to teachers’ professional commitment (Cuervo, 2025).

### 2.2 Teacher commitment in rural contexts

To retain teachers in challenging rural environments and sustain long-term social sustainability, cultivating intrinsic teacher commitment plays a crucial role (Khumalo, 2019). Teacher commitment refers to the degree of psychological attachment teachers have to their school organization and teaching profession, serving as a key indicator for predicting teacher retention, work engagement, and student academic achievement (Meyer and Allen, 1991; Moses et al., 2021). A classic theoretical framework in this field is the Three-Component Model (TCM) proposed by Meyer and Allen (1991), which categorizes commitment into affective commitment (the “willingness to stay” based on emotional attachment), normative commitment (the “should stay” based on a sense of obligation), and continuance commitment (the “need to stay” based on cost–benefit analysis). Although this model originated in a Western context, cross-cultural studies have validated its applicability in East Asian cultural settings, while emphasizing the necessity of culturally adapting measurement tools for specific applications (Lee et al., 2001).

In the context of rural education, existing research has identified various antecedent variables influencing teacher commitment. At micro and meso levels, evidence suggests that transformational leadership behaviors within schools, combined with trust among colleagues, significantly enhance teacher engagement (Khumalo, 2019). Regarding cognitive development, commitment is viewed as a dynamic trajectory extending from early language learning experiences to professional practice, shaped by both personal historical narratives and the macro-policy environment (Moodie and Feryok, 2015). Individual psychological resources, such as emotional competence and self-efficacy, also demonstrate a positive correlation with commitment levels (Phaik Im et al., 2023). Notably, the urban–rural dual structure results in differentiated effects of certain motivational factors: in rural areas with relatively underdeveloped material conditions, teachers’ public service motivation exerts a more significant impact on their satisfaction and potential commitment (Jiang, 2022). Moreover, the quality of internships and mentor support during pre-service education are considered critical foundations for future teaching commitment (Wang et al., 2021).

However, while the literature above reveals a number of psychological and behavioral variables, existing research largely focuses on commitment as an individual psychological attribute, often overlooking how unique rural social structures (such as interpersonal networks and local culture) interact dynamically with individual psychology as external ecological forces. As recent research highlights, understanding the professional status of rural teachers requires examining the dialectical relationship between the social structures in which they are embedded and their agency (Sang et al., 2023).

### 2.3 Place-based education as a proactive mechanism connecting ecosystems and commitments

To explain how the ecological environment leads to multidimensional internal commitment, this study views Place Based Education (PBE) as a core mediating mechanism that connects the work environment and community networks of teachers with their commitments. It also serves as a key example of how teachers demonstrate professional agency (Priestley et al., 2023; Harmon and Johnson, 2025). When teachers face resource scarcity and potential social exclusion in rural areas, PBE offers a practical pathway for them to proactively reshape their ecological environment. By integrating the local environment, cultural heritage, and community resources into teaching, this approach is not only a strategy for instruction but also a process of empowerment. It allows teachers to establish autonomy within a specific context and combat burnout (Greany et al., 2025). Research utilizing visual narratives indicates that when teachers deeply explore their local environments, they can construct a place-based teacher identity. This identity helps them overcome initial feelings of alienation from the rural environment while fostering deep professional and emotional connections with the local community. Thus, the rural area is no longer merely a passive workplace. Instead, it becomes a place of profound professional significance that enhances their sense of belonging (Kim and Uysal, 2025). As a proactive practice, PBE promotes positive interaction between teachers and the rural social and ecological system. This deep interaction satisfies basic psychological needs such as agency and a sense of belonging, which directly fosters a profound sense of professional meaning and teaching efficacy (Riley et al., 2024; Delaune et al., 2025).

Therefore, viewed from an ecological perspective, the teacher agency stimulated by PBE is not an isolated personal trait, but an ecological phenomenon that arises from ongoing interaction (Priestley et al., 2023). When teachers engage in place-based curriculum design through collaborative inquiry, their agency receives significant support (Leijen et al., 2024). However, within a centralized education system, teacher agency is often suppressed in the absence of corresponding belief support and institutional space (AlAjmi and Alsaleh, 2023; Biesta et al., 2015). Hence, PBE serves as more than just an instructional method. It functions as a key practical approach that helps educators overcome environmental constraints within rural education (Greany et al., 2025; Kim and Uysal, 2025). Through this practice, teachers can effectively internalize the external support provided by the rural ecosystem, which ultimately translates into a stable and lasting professional commitment.

### 2.4 Theoretical lens: integrating ecological systems theory and the three-component commitment model

**Figure 1:**
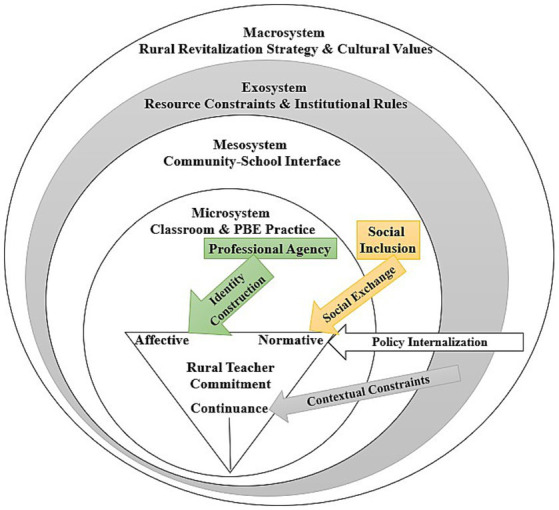
The ecological-commitment analytical framework.

Based on the preceding discussions concerning social sustainability, teacher commitment, and PBE, this study constructs an integrated theoretical framework that analyzes the mechanisms through which the professional commitment of rural teachers in China emerges and evolves (see Figure 1). This framework employs Bronfenbrenner’s Ecological Systems Theory (EST) as the environmental foundation (Bronfenbrenner, 1992; Meyer and Allen’s, 1991). Three-Component Commitment Model (TCM) to delineate psychological outcomes. The advantage of EST lies in its capacity to reveal how factors across multiple layers, ranging from the immediate classroom to the broader culture, collectively influence the resilience and sense of belonging of teachers (Ren et al., 2024; Poole, 2024). This theoretical perspective aligns closely with the broader objective of social sustainability in rural areas, which serves as the core focus of this research.

In the specific context of rural education in China, the ecological system defined in this study comprises four levels. The microsystem refers to the immediate settings where teachers interact directly, namely the classroom and school, where the climate and teaching conditions directly affect self-efficacy (Duan et al., 2024). The mesosystem involves interactions between the school and local community, families, and colleague networks, forming a unique rural social space (Reid et al., 2010). The exosystem encompasses external structures in which teachers do not directly participate but are constrained by, such as evaluation systems and salary policies (Español et al., 2025). Finally, the macrosystem refers to the broader urban–rural dual structure and cultural values (Wen et al., 2023).

The Ecological-Commitment Analytical Framework proposed in this study (Figure 1) follows an “outside-in” logical pathway, viewing commitment as a dynamic outcome of interactions between the individual and multiple ecological levels. First, the macrosystem and exosystem constitute the institutional background and structural constraints for rural teacher retention. Macro-level government policies and mainstream cultural values influence teachers through policy internalization (Jiang, 2022). When teachers identify with national development goals, this macro-narrative transforms into a personal sense of professional mission, serving as a macro-level source of normative commitment (Moodie and Feryok, 2015). However, resource scarcity and institutional regulations (such as salary systems and staffing quotas) within the exosystem often act as stressors (Español et al., 2025), directly impacting teachers’ continuance commitment based on cost–benefit considerations. If the exosystem offers insufficient support, teachers may face survival pressure, necessitating deeper meso- and micro-level mechanisms to provide buffering and motivation.

Under this context, this study identifies two key mediating mechanisms linking the ecological environment to psychological commitment, aiming to clarify how commitment is achieved through specific ecological interaction dynamics (Moodie and Feryok, 2015).

First, social inclusion serves as a bridge connecting the mesosystem to normative commitment (Meyer and Allen, 1991; Cuervo, 2025). This refers to the degree of acceptance and trust teachers receive from the local community and their colleagues. High levels of social inclusion can inspire a sense of moral obligation, thereby strengthening normative commitment. The unique solidarity and social recognition found in rural areas provide a foundation for this commitment, and trust among colleagues has been proven to be a key mediator in promoting professional development (Yao et al., 2024). In contrast, if dual marginalization occurs where teachers are treated as “outsiders” by both urban and rural communities, social exclusion undermines the social exchange basis required to generate normative commitment (Yan and Dai, 2025).

Second, professional agency acts as a link connecting the microsystem to affective commitment (Yao et al., 2024). This refers to teachers’ ability to act according to their beliefs in teaching practice, particularly when implementing PBE (Riley et al., 2024; Delaune et al., 2025). When teachers construct a place-based identity and gain teaching autonomy through PBE, they establish deep emotional connections with the school, promoting the generation of affective commitment (Jaikrasen and Ketsing, 2025; Kim and Uysal, 2025). Furthermore, as an active practice that extends beyond school boundaries, PBE encourages teachers to participate in meaningful interactions with the local community, which represents the mesosystem of their environment. This deep geographical connection effectively reinforces the social inclusion mentioned earlier, and in turn, fosters the normative commitment of teachers toward rural society (Leach and Bradbury, 2024). However, if leadership styles restrict the space for exploration, affective commitment may erode due to professional stagnation (Liu and Zhang, 2024).

The integrated framework discussed above reveals that rural teacher retention is not the result of a single factor but depends on whether the ecosystem provides effective mechanisms, namely, agency achieved through PBE and the social inclusion achieved through trust, to foster specific commitment components. Conversely, inhibiting factors within the ecosystem, such as exclusion and constraints, lead to commitment loss.

## 3 Methodology

### 3.1 Research design

This study employs Interpretative Phenomenological Analysis (IPA) as its core research design, aiming to explore the profound psychological experiences and meaning-making processes of rural Chinese junior high school teachers regarding changes in their professional commitment. The choice of IPA is primarily based on its focus on lived experience. This approach aligns with the study’s goal of moving beyond single-dimensional causal explanations, such as solely attributing changes to salary, to uncover the complex psychological mechanisms behind teachers’ decisions to leave or stay in their profession. As scholars have emphasized in guidelines for applying IPA, this method is particularly suitable for revealing how participants construct self-identity and professional meaning through linguistic narratives within a specific socio-cultural context (Willis and Harvey, 2025). Additionally, studies exploring educators’ implementation of teaching methods confirm that IPA effectively captures the subtle emotional fluctuations and cognitive restructuring processes experienced by educators when facing professional challenges (Delany et al., 2025). Although IPA typically involves a smaller sample size, it pursues idiographic nuance to reveal theoretically transferable psychological structures through the thick description of individual experiences in specific contexts. This approach proves more effective than large-scale statistical data in explaining the underlying motivations for changes in teacher commitment (Hori et al., 2024).

### 3.2 Participants and sampling strategies

Considering the unique socio-ecological environment of rural teachers, this study carefully accounted for the complexity of the rural context during sample selection. First, the study explicitly defined the context as “typical underdeveloped rural areas with population outflow”, distinguishing it from economically developed or suburban rural areas (Gao et al., 2018). Teachers working in these regions face severe resource scarcity and professional isolation, often existing in a doubly marginalized context characterized by exclusion from both urban and rural communities (Yan and Dai, 2025).

Since previous studies have revealed the internal heterogeneity of rural teacher groups and highlighted significant differences in their agency within resource-scarce environments (Palacios Mena and Jiménez, 2024), this study adopted a “Maximum Variation Sampling” strategy. This approach aims to illuminate core phenomena by comparing extreme cases (Ayres et al., 2003). To ensure that these differences were meaningful and to avoid remaining at a superficial level established in advance, this study used a specific logic that involved controlling baseline variables while maximizing characteristic variables during the selection of participants. First, regarding control variables, this study selected four junior high school homeroom teachers as research subjects. Researchers specifically targeted young teachers who were in the early stages of their careers. This decision strictly controlled for variables related to teaching experience and job responsibilities. It also reflected the fact that teachers at this stage often rely more on external support systems than those with more experience (Sun and Huang, 2024). Second, after establishing this controlled baseline, we systematically maximized the characteristic differences within the sample. Researchers used the final decision to stay or leave as the core axis for comparison. Participants were selected from two extreme groups: one group consisted of “stayers” (using the pseudonyms Xinyu and Minhua) who maintained high professionalism and a willingness to stay even under harsh conditions. The other group consisted of “leavers” (pseudonyms Yile and Tingting) who ultimately chose to leave because of a breakdown in commitment, despite having initial enthusiasm and excellent teaching abilities. Beyond this core axis, researchers further increased sample diversity by including different genders, teaching disciplines, and personal backgrounds such as rural origin or urban migration history (see Table 1). This rigorous measure ensured that, within the limited sample size required for IPA, the study not only eliminated interference from generational or occupational factors but also captured the different pathways of interaction between psychology and ecology among individuals facing similar rural pressures.

**Table 1:** Participants’ information.

Name (pseudonym)	Group	Gender	Age	Origin	Subject	Years in rural
Xinyu	Stayer	F	24	Local	Politics	3
Minhua	Stayer	F	29	Outsider	Chinese	6
Yile	Leaver	M	27	Outsider	Math	5
Tingting	Leaver	F	28	Local	Chinese	6

It is important to emphasize that although this study uses the terms stayers and leavers in its analytical framework, this does not represent a rigid or simplistic binary classification. This research deeply recognizes that the psychological engagement of teachers is a dynamic process that fluctuates over time. As Moodie and Feryok (2015) noted, both the individuals who stay and those who depart experience conflicting emotions and temporary periods of stagnation. For example, teachers who ultimately stay also experience strong urges to leave when they face extreme pressure at the micro level. Meanwhile, those who depart often waver between commitment and leaving before they make their final decision. Therefore, this classification mainly reflects the behavioral choices they make at the end of the period examined in this study. The core purpose of this research is to explore how these dynamic psychological changes ultimately form distinctly different trajectories of commitment through various ecological interactions.

### 3.3 Data collection and analysis

Data collection was primarily conducted through semi-structured interviews and document collection to ensure the richness and multi-source corroboration of the data. The interviews were organized into two rounds. The first round focused on life history, which allowed us to trace the initial motivations and early career aspirations of the teachers as they entered the profession. The second round explored critical incidents, focusing on specific moments that led to significant changes in teacher commitment. Although these two rounds appeared separate, they were closely linked at the analytical level. After the first round, the research team immediately performed preliminary transcription and exploratory analysis, which helped identify key interaction patterns in the early experiences of the teachers. These emerging themes then directly informed the design of the second round. By using personalized cues from the initial analysis, the second round focused on the specific moments that caused qualitative shifts in teacher commitment. The interview outline integrated the EST and the TCM. For example, based on the macro and meso systems within EST, researchers developed questions about community integration and how teachers perceive policy pressure. At the same time, researchers used the TCM to design questions about emotional dependence, professional responsibility, and the perceived costs of leaving.

To enhance research validity, researchers followed methodological suggestions regarding data analysis triangulation in case studies (Schlunegger et al., 2024) and extensively collected Supplementary material related to the interview content. These materials included emails (and related recollections), lesson plans, and parent-teacher communication records. During the phase of refining themes, researchers compared and analyzed this textual evidence alongside the interview transcripts. For example, while analyzing the theme of Agency Deprivation for Tingting, a teacher who stayed behind, researchers compared her submitted lesson plans with records of communication between home and school. It is also found that the strict and mechanical requirements for the format of the lesson plans objectively supported the formalistic constraints that she described during her interview. Conversely, when analyzing Xinyu, another teacher who stayed behind and gained autonomy through PBE, the local teaching materials she collected provided direct and objective evidence of the professional agency at the micro level that she mentioned in her interview. This evidence significantly improved the credibility of the research findings.

Data analysis strictly followed the standard procedures of IPA. It combined strategies of integrating “within-case analysis” and “cross-case analysis” (Ayres et al., 2003) to generate themes with universal explanatory power while preserving the unique context of individual cases.

The first stage involved initial coding within each case. Researchers read each participant’s interview line by line and performed open coding on the raw data, focusing on descriptive, linguistic, and conceptual annotations. This process aimed to capture the participants’ most original expressions of experience without immediately applying existing theoretical frameworks. For example, when analyzing Tingting’s interview, we carefully recorded her emotional responses to the work environment as shown in Table 2.

**Table 2:** Example of initial coding process.

Participant	Raw data	Initial code
Tingting	*My supervisor directly criticized me by name in a meeting, saying, ‘This will not be on the exam, stop trying those fancy tricks, just follow the rules.’ Since then, I’ve been spending every day preparing lessons… like a robot in a factory*	Innovation rejected; Job monotony; Lack of autonomy.

The second stage focused on the refinement of emergent themes. In this phase, researchers summarized and abstracted the scattered initial codes from the first stage, transforming them into conceptual clusters with greater psychological or sociological significance. This process involved elevating specific behavioral descriptions to abstract psychological states or social interaction patterns, thereby forming an independent sub-theme structure for each case (see Table 3).

**Table 3:** Example of developing emergent themes.

Participant	Initial codes	Emergent themes
Xinyu	Local resource utilization	PBE implementation
Minhua	Community service exchange	Social integration strategy
Yile	Unfair evaluation	Organizational injustice and exclusion
Tingting	Suppression of innovation	Deprivation of agency

The third stage was cross-case synthesis. After completing the independent analysis of all cases, we juxtaposed and compared the themes of “Stayers” and “Leavers.” The core task of this stage was to identify divergence points in how different cases responded to similar structural pressures, such as workload. By comparing the distinct psychological pathways of teachers in the two groups when dealing with professional setbacks, researchers identified the key mechanisms leading to the maintenance or breakdown of commitment, ultimately forming the core findings of this study as shown in Table 4.

**Table 4:** Example of cross-case synthesis strategy.

Super-ordinate theme	Sub-theme	Evidence from stayers	Evidence from leavers
Social inclusion (meso)	Community and colleague relations	Minhua: Actively integrated and gained “insider” status.	Yile: Encountered invisible boundaries and was excluded due to outsider status.
Professional agency (micro)	Teaching autonomy and PBE	Xinyu: Granted permission to conduct agricultural research, gaining a sense of achievement.	Tingting: Innovation suppressed, feeling a “stagnant” rigidity.
Systemic justice (exo)	Evaluation and reward	Minhua: L ow salary but high self-efficacy.	Yile: Encountered nepotism and unfair evaluation.

## 4 Findings

### 4.1 The macro-exo interface: mission internalization vs. structural injustice

Macro-level national policies and exo-level school management systems together constitute the institutional context of teacher commitment. The study demonstrates that despite generally low salaries, when teachers are able to transform macro-level educational policy goals into personal professional missions and achieve a high sense of self-efficacy, their affective commitment is significantly enhanced. Conversely, when deviations occur in school management implementation, such as nepotism in promotion and evaluation systems, resulting in a lack of organizational justice, teachers’ commitment is placed at risk of collapse (Ghanizadeh et al., 2025).

Minhua exemplifies the concept of mission internalization. Although the salaries of rural teachers under macro-level policies are not absolutely competitive, Minhua has built a strong psychological contract by internalizing policy discourses, such as the “Special Post Program” (Li and Xue, 2021), viewing them as the realization of her personal values. She values not only material rewards but also the sacred moral task bestowed upon rural teachers by national policies. In the interview, she mentioned how she utilizes this sense of macro-level mission to offset the triviality of her daily work:

*When I first came here, I did feel that the conditions were tough and the salary wasn’t high. But every time I saw news about the country revitalizing rural education, I felt that I wasn’t just doing a job, but doing something very sacred… This sense of value, of being needed by the country and society, is something that money can’t buy (Minhua, March 26, 2024).*

Furthermore, student feedback serves as a major source of Minhua’s self-efficacy. In her view, the growth of her students provides a high psychological reward. She mentioned receiving a thank-you letter from a student admitted to high school, which became a key force supporting her continued dedication.

*The letter read, ‘Dear teacher, you changed my destiny and showed me that there is a wider world beyond the mountains.’ I was so moved that I cried when I read that sentence. The salary is indeed low, and I can’t buy all sorts of luxury goods like my classmates who work in the city, but this letter made me feel that what I’m doing is priceless (Minhua, April 28, 2024).*

In contrast to Minhua’s positive transformation, Yile encountered serious structural injustice within the external system. As a key teacher with outstanding teaching abilities, he originally held high hopes for the school’s promotion system. However, the school management prioritized nepotism over merit in implementing evaluation policies. This distorted implementation of the system severely damaged his psychological contract. As a core staff member, Yile led the top class to achieve the best high school entrance examination results in the entire town for three consecutive years. According to custom, the “Outstanding Teacher” title, which is directly linked to professional title promotion, should have been awarded to him. However, in the most recent selection, this honor was given to an older teacher with mediocre teaching performance who was a local resident and a relative of the school’s leadership. This incident became the final straw that broke Yile’s commitment, strengthening his “leaver” identity:

*The principal said to me, ‘Yile, you’re still young, you need to gain more experience, Teacher Cheng is about to retire, we need to take care of him.’ That one sentence completely shattered all my illusions… In this society of personal connections, relationships always outweigh ability… No matter how hard you try, it’s useless because the rules are made for outsiders (Yile, June 30, 2024).*

Yile’s experience profoundly reflects how the “differential mode of association” (hierarchical relational structure) in rural societies erodes the fairness of modern school management. When systemic injustice negates teachers’ professional efforts, normative commitment loses its foundation (Meyer and Allen, 1991).

In comparison, two local teachers, Xinyu and Tingting, demonstrated a more compromising attitude regarding the interaction between the macro and external systems. Although Xinyu was also aware of salary limitations, she considered the job security provided by her permanent position the greatest benefit of the macro policy. This continuance commitment, based on realistic considerations, allowed her to remain content within the system. Tingting, on the other hand, was caught in a weary struggle, feeling exhausted by endless administrative checks and bureaucratic formalities within the system. In an interview on June 30th, she helplessly described the predicament of being overwhelmed by administrative tasks: “*We’re busy filling out all sorts of forms every day, leaving very little time for lesson preparation… Although we have to do it to keep our jobs.*” This indicates that while the administrative burden has not yet reached the point where she would consider quitting, it has significantly eroded her job satisfaction, leaving her in an unstable psychological state.

### 4.2 The mesosystem: social inclusion vs. the invisible border

The mesosystem involves the interaction of teachers with the local community and school social networks. As Social Exchange Theory posits, reciprocal social relationships serve as an important link in maintaining an individual’s commitment to an organization (Yan and Dai, 2025). This study found that in the mesosystem of rural communities, characterized by close-knit relationships, the quality of interaction between teachers and the community (as well as their colleagues) directly affects their affective commitment. For teachers from outside the area, the ability to cross the invisible boundary between “locals” and “outsiders” determines whether they become “insiders” in the community or marginalized “outsiders.”

Xinyu, as a native of the village, is naturally embedded in the local social network. This “insider” status allows her to easily gain the trust and support of parents and the community, forming a positive cycle of social exchange. Such inclusion and support from the rural community have greatly enhanced her sense of belonging and affective commitment.

*In this village, I’m not just a teacher, but also someone’s granddaughter, someone’s niece… When I go on home visits, the parents never treat me as an outsider… This kind of kinship and closeness makes me feel especially warm and secure (Xinyu, March 26, 2024).*

Conversely, Yile, a teacher from outside the area, experienced strong exclusion within the close-knit rural community. Although his teaching performance was impeccable, in informal social networks, the language barrier and differences in regional culture constituted an insurmountable invisible boundary. This sense of social isolation prevented him from establishing a true sense of belonging. He stated in the interview:

*The hardest part isn’t the hard work, but the loneliness of not being able to fit in. In the office, everyone chats in the local dialect, and I’m left out like a deaf person… That loneliness isn’t about nobody talking to you, but about feeling out of place in a crowd, constantly reminded that ‘you’re an outsider’ (Yile, June 2, 2024).*

Yile’s experience confirms the difficulty of marginalization that outsiders often face in rural social structures (Yan and Dai, 2025). This persistent social exclusion not only weakened his sense of belonging but also led him to feel that his efforts were not commensurate with the rewards, thus undermining his normative commitment.

On the other hand, Minhua, also an outsider, presented a different situation. Although she initially faced cultural and language barriers, her background in social work led her to adopt a proactive social integration strategy. She did not confine herself within the campus walls but utilized her free time to visit villages, reading letters for elderly villagers whose grandchildren were working elsewhere, and providing psychological counseling to families of troubled students using her professional knowledge. This community service, extending beyond her teaching duties, triggered positive social exchange. Villagers began to reciprocate in simple ways, allowing her to gradually build deep emotional connections in this unfamiliar place. In the interview, Minhua emotionally recalled the details of her interactions with the villagers:

*When I first arrived, I couldn’t understand the local dialect, and I felt very lonely… Later, when I went on home visits, I helped the elderly villagers read letters sent by their grandchildren who were working away from home. Gradually, a bag of home-grown vegetables or a few eggs would often appear at my dorm room door… At that moment, I suddenly felt that this was not only my workplace but also my home. That feeling of being needed and accepted made me feel that staying here was meaningful (Minhua, March 26, 2024).*

This social inclusion from the community greatly strengthened Minhua’s affective commitment, offsetting the hardships of rural life.

### 4.3 The microsystem: professional agency vs. stagnation

At the micro-level of the classroom and teaching, whether teachers can exercise professional agency is crucial in determining their continued commitment (Kim and Uysal, 2025). When teachers can innovate teaching methods based on local contexts, their sense of professional meaning significantly increases. Conversely, if teaching activities become mechanized and rigid, teachers are prone to professional burnout.

Xinyu, a local teacher, deeply understood rural students’ resistance to dry textbooks. Instead of simply following the curriculum, she fully utilized local resources by moving her classroom to the fields. She organized students to conduct field research at a local fruit farm, encouraging them to interview farmers about e-commerce sales. This PBE not only activated the students’ learning interest but also allowed Xinyu to discover her professional direction in cultivating talent for her hometown. The agency gained through building a local identity (Kim and Uysal, 2025) became her psychological capital against the low-wage environment.

*“That day at the farm, the eyes of several boys who usually slept in class lit up. At that moment, I realized that education is not just about making students memorize books, but also about letting them see hope in life… Although the salary here is not high, seeing the children feel that studying is useful, I feel that I have value on this platform… This sense of accomplishment is something that working in big cities cannot provide” (Xinyu, April 28, 2024).*

Furthermore, the Supplementary material for the local curriculum design that Xinyu provided, such as the orchard survey worksheet and screenshots of electronic commerce data collected by students, clearly show how she transformed broader policies and local resources into teaching actions at the micro level. These objective documents powerfully support the agency she gained by building a sense of local identity (Kim and Uysal, 2025). This indicates that her autonomy does not only exist within her personal stories but is also put into practice through real teaching activities. In this way, it becomes a form of psychological capital for her as she works in an environment where income is low.

However, another local teacher, Tingting, experienced a setback in her agency within the microsystem. She had enthusiastically attempted to conduct a “local reading class”, guiding students to read essays about rural life in an effort to increase their identification with local culture. However, this initiative deviated from the standardized exam-oriented approach and was severely criticized by the teaching research group leader. This top-down administrative pressure deprived her of teaching autonomy.

*“My supervisor directly criticized me by name in a meeting, saying, ‘This won’t be on the exam, stop trying those fancy tricks, just follow the rules.’ Since then, I’ve been spending every day preparing lessons, teaching classes, and grading assignments, like a robot in a factory… I feel like my life is a stagnant pool, without any ripples… I feel like I’ve already seen what I’ll be like at sixty before I even turn thirty” (Tingting, June 2, 2024).*

Additionally, the recent lesson plans and records of communication between parents and teachers that were submitted as Supplementary data provided triangulation for the feeling of administrative oppression that Tingting described during her interview. An analysis of the texts confirmed that she was required to complete her lesson plans strictly according to a template that was highly standardized. Meanwhile, her weekly records of communication with parents were quantified into fixed assessment items, which left almost no physical space for personalized instructional design. This textual evidence further supported the account provided by Tingting as it clearly revealed how institutionalized rules that suppress the innovative spirit of a teacher can quickly lead to a loss of professional meaning and a strong desire to quit.

## 5 Discussion

**Figure 2:**
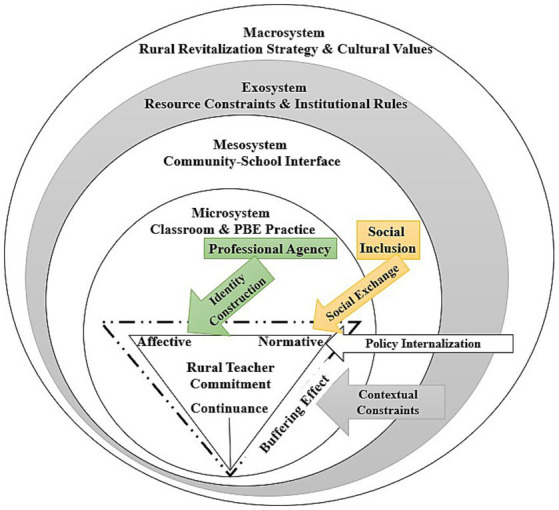
Ecological pathway of retention: the buffering effect.

**Figure 3:**
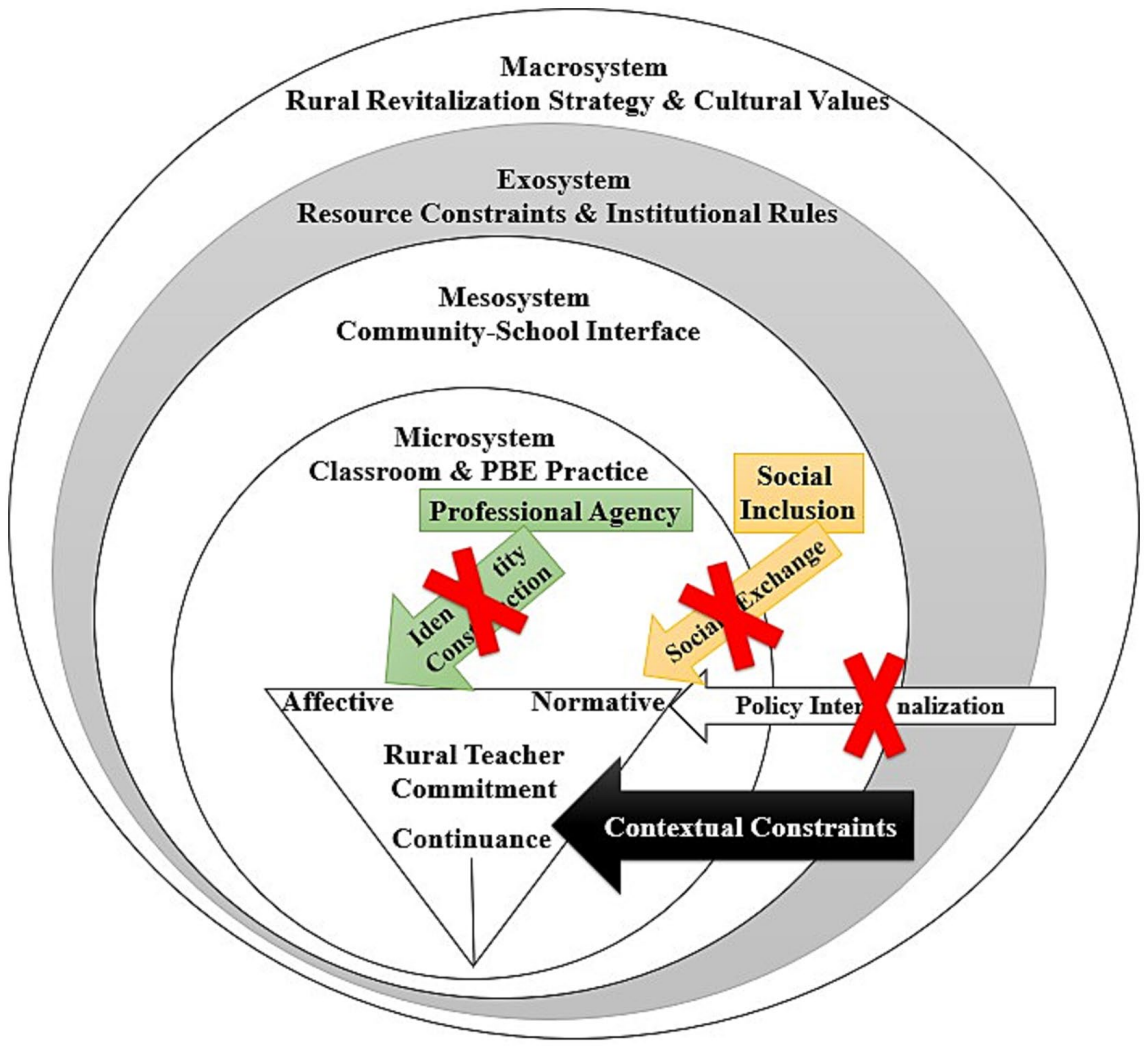
Ecological pathway of attrition: the ecological stagnation.

This study constructs a “Comparative Ecological Pathway Model of Rural Teacher Commitment” (see Figures 2, 3) based on the integrated analytical framework established earlier (Figure 1). This model systematically addresses the study’s two core inquiries by integrating multi-level interactive relationships: it explains why teachers in similar environments follow vastly different career trajectories (RQ1) and delineates how the ecosystem shapes commitment through specific mechanisms (RQ2). The findings indicate that retention or turnover is not determined solely by individual psychological traits but by a complex interaction among macro-level policies, school systems, community environments, and individual agency.

### 5.1 Mechanisms of commitment differentiation: the buffering effect vs. ecological stagnation

To address RQ1 regarding the differentiation in career trajectories between “stayers” and “leavers,” this study compares the experiences of Minhua and Xinyu (stayers) with those of Yile and Tingting (leavers) to propose two distinct mechanisms: the buffering effect and ecological stagnation.

Figures 2, 3 serve as dynamic expansions and comparative applications of the framework presented in Figure 1. While Figure 1 depicts the macro, exo, meso, and micro systems as nested static structures, Figures 2, 3 reorganize these systems into dynamic processes explaining retention and attrition, respectively. The findings reveal that all interviewed teachers faced resource constraints from the exosystem, particularly low salaries and heavy workloads, which aligns with previous research on the living conditions and job satisfaction of rural teachers (Zhi et al., 2025; Gao et al., 2025). However, the key variable determining commitment persistence was not the pressure from the exosystem itself, but rather whether effective protective mechanisms were formed within the meso and micro systems.

For Minhua and Xinyu, the retention path in Figure 2 demonstrates a virtuous cycle. Despite objective pressures such as low salaries in the exosystem, professional agency at the micro level and social inclusion at the meso level together constituted a strong buffering effect. This finding expands upon the concept of teacher resilience, suggesting that resilience is not merely an individual psychological characteristic but the result of support from a multi-layered ecological system (Sun and Huang, 2024; Vieira et al., 2024). Specifically, Minhua activated agency in the micro system through PBE practices, while Xinyu gained support from the mesosystem by integrating into the local community. Through effective social exchange and identity construction mechanisms, these two forces successfully resisted the negative impact of the exosystem and transformed macro-system rural revitalization policies into a personal sense of mission (Jiang, 2022), thereby maintaining high levels of affective and normative commitment (Meyer and Allen, 1991; Raymond and Gabriel, 2023).

Conversely, the attrition path in Figure 3 explains the departure of Yile and Tingting, where the ecosystem acts as a negative bottleneck. Due to a lack of meso-level social inclusion (exemplified by the dialect and cultural exclusion faced by Yile) and the deprivation of micro-level agency (exemplified by the innovation stagnation faced by Tingting), these teachers lost the buffering mechanisms necessary to cope with external systemic pressures (AlAjmi and Alsaleh, 2023; Yan and Dai, 2025). In this situation, low pay and unfair performance evaluations (exosystematic constraints) directly breached their psychological defenses, leading to a breakdown of commitment. This path model demonstrates that when the ecological support system fails, macro-level policies cannot be internalized, and external systemic pressures become the decisive factor overwhelming teachers (Español et al., 2025).

### 5.2 Microsystem dynamics: agency, PBE, and affective commitment

Addressing the second research question concerning ecosystem interaction mechanisms—specifically how micro-interactions shape affective commitment—this study found that classrooms and teaching practices serve not merely as venues for fulfilling duties but as core arenas for teachers to construct professional identity and affective commitment. The pathway in Figure 1, extending from microsystem to self-efficacy and subsequently to affective commitment, is fully validated by the findings (Figures 2, 3).

Minhua’s case demonstrates that PBE practices, which integrate local resources into teaching, significantly enhanced her professional agency. This agency not only fostered a sense of direct contribution to students’ growth but also generated a feeling of indispensability, thereby deepening her affective commitment. This finding resonates with discussions on teacher identity and emotional investment, suggesting that when teachers embody personal values in their teaching and construct a place-based identity, their commitment levels increase significantly (Jaikrasen and Ketsing, 2025; Kim and Uysal, 2025). Furthermore, high self-efficacy has been proven to act as a buffer against burnout in inclusive or complex educational environments, protecting teachers’ affective commitment from depletion (Phaik Im et al., 2023; Vieira et al., 2024).

However, microsystem also present substantial challenges to agency. For Tingting, the pressure of standardized testing and rigid instructional management deprived her of classroom autonomy, leading to the innovation stagnation depicted in Figure 3. When teachers cannot exercise agency within the microsystem, the classroom devolves into an alienated workplace rather than a space for meaning-making. This finding supports research on the limitations of teacher agency in centralized educational systems, where a lack of professional autonomy and a restrictive leadership style are significant causes of teacher innovation stagnation and burnout (AlAjmi and Alsaleh, 2023; Liu and Zhang, 2024). Simultaneously, such technical pressure and hindered professional development are directly linked to diminished work commitment (Ghanizadeh et al., 2025).

Furthermore, while examining the regulatory role of the microsystem, it must be acknowledged that the professional initiative that teachers exhibit is not entirely shaped by the external environment. Research has found that even in micro environments that face similar resource constraints, the coping strategies of teachers still show significant diversity (Sun and Huang, 2024). This indicates that individual psychological characteristics which exist beyond the ecosystem play an important regulatory role. As Moodie and Feryok (2015) points out, the engagement of a teacher is deeply rooted in their personal history and narrative. Individuals who have specific memories of early rural life or higher emotional capital (Phaik Im et al., 2023) are often able to use their internal psychological resources (Leach and Bradbury, 2024). These individuals demonstrate resilience that goes beyond environmental constraints, even in ecosystems that are highly challenging. Therefore, the ecosystem is not so much a structure that determines commitment in a one-sided way. Instead, it is a field that interacts with individual traits in a dialectical manner. The continuous interplay between the internal motivation of an individual and the micro ecological environment forms the complete dynamics of how affective commitment is generated.

Based on this mechanism of the dialectical interaction between the microsystem and individual psychology, this study argues that the key to maintaining commitment of rural teachers lies in more than just reducing objective administrative burdens. It also involves creating a flexible space for curriculum innovation within the microsystem. Specifically, PBE should not be viewed merely as a teaching method, but should be seen as an empowering mechanism that embraces the diversity of individuals (Greany et al., 2025). By providing this space, teachers who have different personal histories and different levels of psychological capital can find entry points where they can transform their internal motivation into professional practice. Thus, when they face complex teaching situations, they can continuously reshape their deep affective commitment through mechanisms of self-efficacy (Priestley et al., 2023; Harmon and Johnson, 2025).

### 5.3 Meso-level social exchange: social inclusion as a key determinant of retention

Regarding the meso-level interaction mechanisms explored in RQ1, this study confirms the central role of social exchange in shaping normative commitment. Figure 1 emphasizes that the meso-level system directs influence toward normative commitment via social exchange mechanisms. The data from this study strongly support this theoretical hypothesis and reveal the decisive role of social inclusion. Xinyu’s ability to overcome initial difficulties and develop a strong intention to stay was largely attributable to the deep emotional connections she forged with the local community and parents. These connections transcended formal working relationships, forming a social exchange contract based on reciprocity and trust. According to social exchange logic, when teachers perceive a sense of solidarity and respect from the community, they develop a moral obligation to remain (Meyer and Allen, 1991; Cuervo, 2025).

In contrast, Yile’s case demonstrates how the liability of foreignness hinders the formation of commitment at the meso-level. As a teacher from outside the region, language barriers and cultural differences precipitated a dilemma of social exclusion, depicted as an ecological stagnation in Figure 3. The lack of community support not only severed the source of normative commitment but also exacerbated the isolation of rural life. This finding indicates that the retention issue of rural teachers cannot be confined solely within school walls but must be examined within the broader community ecosystem (Reid et al., 2010). Such double marginalization, being perceived as an outsider in both urban and rural settings, undermines the teacher’s psychological contract (Yan and Dai, 2025). The failure to establish effective home-school-community interaction interfaces results in teachers becoming emotionally disconnected from the local environment, thereby accelerating turnover (Sun and Huang, 2024).

### 5.4 Macro-exo discrepancies: policy internalization vs. institutional constraints

At the levels of the macro and exo systems, this study reveals a misalignment between national strategic intentions and grassroots institutional implementation. The macro system in Figure 1 (the rural revitalization strategy) aims to inspire a sense of mission among teachers through policy internalization. The findings indicate that retained teachers, such as Minhua and Xinyu, successfully internalized this macro discourse into their personal career goals, believing they were cultivating talent for the future of rural areas. This infusion of macro values provided a spiritual foundation for their commitment that transcended material interests, confirming the unique role of Public Service Motivation in enhancing teacher satisfaction and commitment in rural contexts (Moodie and Feryok, 2015; Jiang, 2022).

However, resource limitations and institutional regulations within the exosystem often conflict with this macro vision. The study found that both retained and departing teachers expressed dissatisfaction with low salaries and unfair professional title evaluations. For Tingting and Yile, the institutional injustice and resource scarcity depicted in Figure 3 constituted a direct push factor. When negative feedback from the external system, such as the imbalance between effort and reward, or the lack of support for educational policy implementation, becomes overwhelming, and buffering mechanisms from the meso and micro systems are absent, lofty macro-level slogans often fail to translate into tangible motivation (Zhi et al., 2025; Español et al., 2025). This finding serves as a warning that relying solely on moral appeals at the macro level is insufficient to maintain the stability of the teaching force. It is essential to address institutional obstacles within the external system, particularly by optimizing salary structures and evaluation systems, to mitigate their corrosive effect on teacher commitment (Gao et al., 2025).

At a deeper level, the interaction between the structural constraints of the external system and the retention of individuals does not always follow a simple linear relationship. As the research findings reveal, in addition to deep affective and normative commitments, the retention of teachers may also stem from a continuous commitment that is based on considerations regarding costs and benefits (Meyer and Allen, 1991). For example, the practical consideration by Xinyu regarding the employment security that extended tenure provides also contributed to her choice to remain in her position. This indicates that commitment in certain dimensions cannot be fully explained by the positive ecological empowerment found within the meso and micro systems. Instead, it is profoundly limited by the rigid constraints of the external environment (Yan and Dai, 2025; Zhi et al., 2025).

On the other hand, within the actual field of rural education, even if some teachers face severe ecological stagnation and fail to establish effective psychological buffers, the harsh realities of the external system may still force them to remain in their original positions. These realities include a lack of alternative employment opportunities, sunk costs, or geographical ties to their families. This phenomenon of being detached from the ecological support system further highlights the complex tension present in the interaction between the psychology of teachers and their environment. When the external environment is oppressive and lacks a buffer mechanism, it does not necessarily lead to the turnover of teachers. Instead, it can easily degenerate into a state of passive retention that lacks substantial professional commitment (Moodie and Feryok, 2015). This recognition thereby provides a more comprehensive view of the complex nature of the career trajectories of teachers under broader structural constraints (Wen et al., 2023).

### 5.5 Implications: teachers as agents of rural revitalization

The findings of this study not only concern the career trajectories of individual teachers but also profoundly reflect the crucial role of rural education in the accumulation of agricultural human capital. Against the backdrop of increasingly severe rural depopulation (Wen et al., 2023), retaining teachers like Xinyu and Minhua, who possess PBE capabilities and a strong sense of community, is vital for overcoming the challenges of social sustainability in rural revitalization (Sarfo et al., 2025). These stayers effectively act as bridges connecting students with their local communities by implementing PBE. The study demonstrates that high-achieving rural students are not destined to leave; they often possess a deep attachment to their communities, an attachment that largely depends on their perceptions of the local economy and future prospects (Petrin et al., 2014). Xinyu’s case, in which she led students to explore the agricultural industry, demonstrates that when teachers are empowered to develop local curricula, they do more than teach knowledge. They integrate the United Nations SDGs into local practices through social innovation (Delaune et al., 2025). This type of education cultivates students’ understanding of and affection for rural areas, thereby reserving the necessary human capital for future agricultural modernization.

Conversely, the attrition paths of Yile and Tingting foreshadow a potential double loss of talent. On one hand, highly capable teachers leave due to social exclusion, causing rural areas to lose opportunities to introduce external intelligence and open culture, which exacerbates the disadvantages faced by outsiders (Yan and Dai, 2025). On the other hand, local young teachers with innovative aspirations leave due to institutional rigidity, severing the endogenous driving force for innovation in local education. This attrition represents not only a loss for the education system but also a weakening of the social sustainability of rural communities (Liu and Zhang, 2024). Therefore, protecting teacher commitment is essentially protecting the foundation of future rural human capital. If the ecosystem continues to filter and block resources at every level instead of providing support and relief, rural education will find it difficult to break free from the vicious cycle of exam-oriented education and talent outflow (Wen et al., 2023).

Based on these findings, this study proposes three specific policy recommendations aimed at empowering teachers to become effective agents of rural revitalization through systemic support (Barnes et al., 2024; Shula and Heystek, 2025):

First, regarding the social integration challenges faced by teachers from outside the region, it is recommended that local governments establish an effective channel for integration to promote social inclusion. Simply relying on recruitment policies to bring teachers to rural areas is insufficient; attention must be paid to their social and ecological experiences after joining the profession. Local governments should collaborate with communities to provide specific talent housing for teachers from outside the region, especially professionals in agricultural technology, and regularly organize social events that integrate local culture. This will proactively dismantle the invisible barriers caused by dialects and geographical differences, helping teachers transform from “outsiders” to “insiders” within the community. Simultaneously, specialized psychological support services must be introduced to address the loneliness and cross-cultural adaptation stress often faced by young teachers from outside the region. It is recommended that schools or education bureaus establish a regular psychological counseling mechanism, utilizing interpersonal support to alleviate emotional difficulties in the early stages of teachers’ careers and promote the development of emotional resilience (Bronfenbrenner, 1992). As research suggests, government-funded alternative pathways must be complemented by a comprehensive strategy of continuous improvement in work environments, welfare benefits, and psychological care (Gao et al., 2025) to effectively enhance teachers’ professional adaptability and willingness to stay (Barnes et al., 2024; Zhi et al., 2025).

Secondly, regarding the obstacles to innovation faced by ambitious local teachers, it is recommended that county-level education departments establish local-based educational innovation awards. Educational administrative departments should shift their evaluation focus to encourage teachers to develop local school-based curricula closely integrated with rural revitalization. Guiding students to participate in agricultural practices and rural research should be included as bonus criteria in teacher performance evaluations and professional title promotion, rather than being considered a neglect of duty. By establishing a supportive system structure and governance mechanism, PBE can become a source of inspiration for teacher agency and professional meaning rather than an additional administrative burden (Greany et al., 2025).

Finally, in response to the issue of unfair evaluations prevalent in close-knit communities, it is recommended that rural schools establish a supervisory mechanism involving third parties. Processes involving teachers’ vital interests, such as performance evaluations and promotions, should be managed transparently to prevent the erosion of organizational fairness by clan influence or nepotism. Given that centralized organizational structures often restrict teacher autonomy and lead to passive resistance, building a fair, transparent, and supportive institutional environment is crucial for maintaining teachers’ commitment to professional standards (AlAjmi and Alsaleh, 2023).

## 6 Conclusion

Adopting an ecological systems perspective, this study provides an in-depth analysis of the formation and evolution mechanisms of professional commitment among rural junior high school teachers in China. The results indicate that the psychological decision to stay or leave depends not solely on macro-level policies or material benefits, but critically on whether the teacher’s ecological system supports two fundamental psychological needs: social inclusion at the meso-level and professional agency at the micro-level. The findings reveal that committed teachers construct effective coping mechanisms by rooting themselves in local communities and exerting agency through PBE, actions that generate deep affective and normative commitment. In contrast, those who leave experience ecological stagnation due to social exclusion and inhibited autonomy, leading to burnout and commitment breakdown. This study extends the understanding of teacher retention by highlighting that commitment is an ecological outcome of the interaction between the individual and their environment. It emphasizes that to maintain the stability of the teaching force, the rural school ecology must cultivate multi-layered social and professional belonging, empowering teachers to find meaning and agency within their specific context (Poole, 2024). Only when the ecological system fosters such psychological integration can rural education effectively sustain its most vital resource, i.e., its teachers. This study also moves beyond a simple view of structural determinism (Eteläpelto et al., 2013). Under strict external constraints, such as the dual marginalization between urban and rural areas (Yan and Dai, 2025), some teachers exhibit passive retention. This condition lacks substantial professional engagement, which aligns with the concept of continuous commitment (Meyer and Allen, 1991). Teachers typically remain in this state due to concerns over sunk costs or a lack of alternative employment. As recent studies have revealed, when institutional spaces are highly oppressive, the agency of teachers often easily transforms into compromise or passive resistance (AlAjmi and Alsaleh, 2023). This finding further highlights the complex dialectical tension that exists between the internal psychological dynamics of individual teachers and broader structural constraints.

Although this study revealed the underlying mechanisms of the evolution of rural teacher commitment through IPA, it possesses certain limitations. First, the study’s fieldwork was strictly confined to typical underdeveloped rural areas in China characterized by significant population outflow. While this specific context profoundly highlights the phenomenon of double marginalization under conditions of resource scarcity (Yan and Dai, 2025), it may not fully encompass the living conditions of teachers in economically developed regions or suburban rural areas. Therefore, future research should expand its scope to include comparative analyses across rural areas with varying levels of economic development, thereby enriching our understanding of teacher development within the urban–rural dual structure. In addition, the data in this study were derived primarily from interviews and document analysis. Although a triangulation strategy was employed, the findings may still be influenced by participants’ recall bias. Given that teacher commitment is a dynamic process that fluctuates over time (Moodie and Feryok, 2015), future research could utilize a longitudinal design to more accurately capture the evolving trajectory of the interaction between the ecosystem and teacher commitment across different stages of their careers (Bönke et al., 2024).

## Data Availability

The original contributions presented in the study are included in the article/supplementary material, further inquiries can be directed to the corresponding author.
